# Knowledge, Attitude and Practices of Frontline Health Care Workers to Disaster Risk Management in Private General Hospitals in Addis Ababa, Ethiopia: Multicenter Cross-Sectional Study

**DOI:** 10.4314/ejhs.v33i5.9

**Published:** 2023-09

**Authors:** Bekele Getenet, Woldesenbet Waganew, Desalegn Keney, Aman Yesuf

**Affiliations:** 1 Debre Markos University, Debre Markos, Ethiopia; 2 St. Paul's Hospital Millennium Medical College, Addis Ababa, Ethiopia

**Keywords:** Disaster, risk management, private hospitals, Ethiopia

## Abstract

**Background:**

Disaster is an acute dysfunction of the existing environment that requires external assistance. Although disaster has had a significant impact in Ethiopia, little is known about KAP of frontline HCW on disaster management in private hospitals. Therefore, this study will be a background for future researches and disaster management plan in private health sectors. The study was conducted to assess the knowledge, attitudes, practices and their influencing factors among frontline healthcare workers on disaster risk management in private general hospitals in Addis Ababa.

**Methods:**

The study design was multicenter cross-sectional survey that used structured closed- and open-ended questions. Multi-stage sampling technique was used. The sample size was 270 with a response rate of 98.9%. The study was conducted in frontline HCW of six private general hospitals from July 20-September 30, 2022. Epi-info version 7.0 and SPSS-25 were used for data clearing and statistical analysis. Level of KAP was calculated from the participants' scores of the questions. Associations were done by using bivariate and multivariate logistic regression.

**Results:**

Of the participants, 64% had poor level of knowledge, and 89.10% had poor level of practice while 93.6% had positive attitude. Lack of previous training, inadequate level of practice, and health experience below one year were negatively associated with good level of knowledge. Poor level of knowledge was negatively associated with good practice.

**Conclusion:**

Although the majority of the participants had positive attitude, the mean level of knowledge and practice were poor to properly handle disastrous events.

## Introduction

Disaster is a serious acute deterioration of the functioning of society that overwhelms the capacity of the affected regions to overcome it with their own resources; so external assistance is required (1-4).

Around 1.6 million people have died because of disasters in the world that is approximately 65,000 deaths annually, and trillions of dollars in related damages have been lost, since 1990. Disasters are able to change the appearance of a developing nation suddenly and may collapse their years of achievement (5-7).

Disaster risk management is the knowledge, capacities and organizational systems developed by governments, response and recovery organizations, communities and individuals to effectively anticipate, respond to, and recover from the impacts of likely, imminent, emerging, or current emergencies (2, 8, 9). The health service in disasters is mainly targeted to save lives and maintain the wellbeing of the victims. Disaster response requires adequate human resources in both public and private health sectors. Uninterrupted service flow should be created and maintained from the disaster scene to final treatment centers (2, 8, 9).

Globally, disaster related impact is increased. Around 7348 natural disasters were reported in the world, from which 1.2 million people were died and 4 billion people were affected in 2000-2019. By the end of 2021, the COVID-19 pandemic had killed 2 million people, with continuous growth of infected people and emergence of new variants. Developing countries are significantly affected by disaster, and the risk is being extended with climate change (10-12).

In 2010, outbreaks of infectious diseases were reported in many African countries. In the same year, cholera affected 100,000 people and killed 3000 people while meningitis affected 29,000 people and killed 3000 people (13). Although many reports of disaster losses have been underestimated, African countries have lost around $2.5 trillion in this century (14).

Many parts of human life, particularly in developing countries, are markedly being affected with severe consequences of disastrous situations due to significant distraction and inadequate disaster preparedness, which needs involvement of public and private health institutions (6, 15). To address these problems, the 3^rd^ UN Conference (2015) established the “Sendai Framework for Action” across the disaster risk management continuum of prevention, preparedness, response, and recovery and focused on the resilience of communities, and health and social systems (16).

Disaster risk management needs a scientifically studied interrelated chain between community, government and private organizations. Although few published researches on disaster risk management in communities and public institutions were done in Addis Ababa (AA), there is no published study regarding disaster risk management in private health institutions. Around 40-60% of HCW in private and public hospitals of AA did not know whom to contact during infectious disease outbreak in hospitals, and the majority of HCW were not confident to handle a suspected case of COVID-19 (17).

Hence, this study assessed KAP and its influencing factors of HCW in private general hospitals and to develop recommendations for health facilities, leaders and policy makers on identified gaps. Additionally, this study could be used as a scientific evidence for future studies.

## Methods

The study design was multicenter cross-sectional survey by using structured closed- and open-ended questions. The study was conducted at private general hospitals in Addis Ababa, which is a capital city of Ethiopia and the seat for the African Union (AU). It covers an area of 530 square kilometers and has a population size of 3,048,63, of whom 1,595,968 were females and the rest 1,452,663 were males during the study period. The city is divided into 11 sub-cities and has 49 hospitals. Thirteen are public hospitals. Thirty-six of the city's hospitals are private, from which 21 hospitals serve as general hospitals. The study was conducted from July 20-September 30, 2022. All HCWs in Addis Ababa private general hospitals during the study period were considered as the source population.

The sample size for the study was 270. It was determined by using population correction formula as the source population was below 10,000, and 10% drop out was added.

HCW working in, ED, triage areas, pharmacies, laboratories, and imaging rooms for at least six months were included; while HCW who are working there below 6 months, part-time HCW, and interns were excluded.

Age, gender, educational level, profession, experience, working institution, training, disaster risk management plan, and drill were independent variables. And knowledge on disaster risk management, attitude on disaster risk management, and practice on disaster risk management were dependent variables.

The questionnaire was developed after prior similar studies were reviewed and used with certain modifications (6, 18, 19). Data was collected with structured open- and close-ended items that included seven socio-demographic questions, eleven knowledge related questions, seventeen attitude questions and seven practice related questions.

Data coding and entering was performed using Epi-info 7.0. Frequency and cross-tabulation were used to check for missed values and variables. Statistical analysis was done using SPSS 25. Descriptive analyses and associations were reported and presented in figures and tables.

All data were checked for clarity, completeness and correct recording by the principal investigator. Ethical clearance was obtained from the Ethical and Research Committee of SPHMMC. The hospital administrators were informed about the purpose of the study, anticipated benefits, selection criteria, and data collection procedures. Confidentiality of participants was kept during the study and throughout dissemination of the result.

The following operational definitions are used.

**Frontline HCW**: HCW who are actively involved in patient diagnosis and clinical management

**Drills**: exercises in which health care workers simulate the circumstances of a disaster.

**Good knowledge**: participants who have scored at least 50% on knowledge questions

**Poor knowledge**: participants who have scored below 50% on knowledge questions

**Positive attitude**: participants who have scored above 50% of attitude questions

**Negative attitude**: participants who have scored at least 50% on attitude questions

**Good practice**: participants who have scored at least 50% on practice questions

**Poor practice**: participants who have scored below 50% on practice questions

## Results

**Socio-demographic characteristics of the study participants**: A total of 267 health professionals participated in the study making a response rate of 98.9%. From the total participants, 139(52.1%) were females and 128(47.9%) were males, making the male-to-female ratio of 0.9:1.

More than 2/3^rd^ (69.7%) of the participants were in the age range of 21-30 years, and the mean age of the study participants was 28.99 with SD 5.23. Most of the taken (77.9%) had never taken training about disaster risk management (Table 1).

**Table 1:** Socio-demographic distribution of participants in private general hospitals, AA, July 20-September 30, 2022

Variable		Frequency (n=267)	Percent
Gender	Male	128	47.9
	Female	139	52.1
Age category in years	21-30	186	69.7
	31-40	72	27.0
	41-50	8	3.0
	51-60	1	0.4
Marital status	Married/widowed	111	41.6
	Single	156	58.4
Educational level	Specialist	26	9.7
	Resident	6	2.2
	Master/GP	52	19.5
	BSc	163	61.0
	Diploma	20	7.5
Work experience	Below 1 year	12	4.5
	1-5 years	161	60.3
	6-10 years	79	29.6
	11-15 years	12	4.5
	16-20 years	3	1.1
Previous training on	Yes	59	22.1
DRM	No	208	77.9

AA-Addis Ababa, BSc- Bachelor of Science, GP-General Practitioner, DRM-Disaster Risk Management

**Figure 1:**
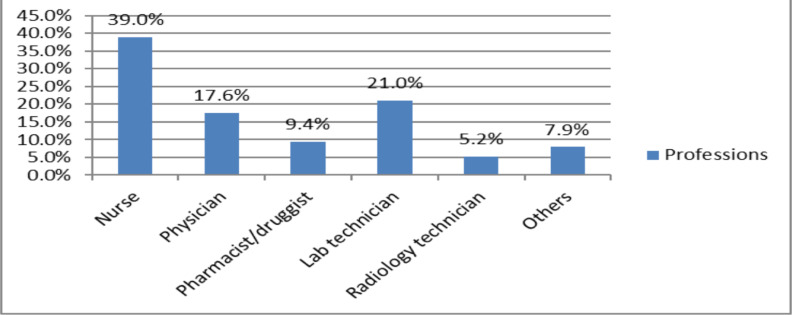
Column graph illustrating professional categories of participants in private general hospitals, Addis Ababa, July 20-September 30, 2022

The majority (39%) of the participants were nurses, followed by laboratory technicians and physicians (Figure 1).

**Level of knowledge towards disaster risk management**: Around 64% of the participants had poor level of knowledge towards disaster risk management. The majority of the participants (70.8%) rated their level of knowledge for disaster risk management as favorable (good and very good) (Table 2).

**Table 2:** Assessment of participants' knowledge on disaster risk management in private general hospitals, AA, July 20-September 30, 2022

Questions	Responses	Frequency	Percent
Meaning of disaster	Correct	184	68.9
	Incorrect	65	24.4
	Don't know	18	6.7
Meaning of DRM	Correct	125	46.8
	Incorrect	119	44.6
	Don't know	23	8.6
Presence of DRM plan in their institution	Know	82	30.7
	Don't know	185	69.3
Site of DRM plan copy	Know	30	11.2 (valid %=36.6)
	Don't know	52	19.5 (valid %=63.4)
Know what should be included in DRM plan	Yes	101	37.8
	No	166	62.2
Contents of DRM plan	Equipment	4	1.5 (valid %=4)
	Evacuation system	3	1.1 (valid %=3)
	Vulnerability assessment	8	3.0 (valid %=7.9)
	All above should be included	86	32.2 (valid %=85.1)
Time of alert status activation	Know	106	39.7
	Don't know	161	60.3
Place of patient evacuation	Know	81	30.3
	Don't know	186	69.7
Meaning of drill	Know	84	31.5
	Don't know	183	68.5
Drill practice in your institution	Seen	87	32.6
	Never seen	180	67.4
First aid provision	Immediately (at the scene)	248	92.9
	In hospital	19	7.1
First aid rescuer	Only health worker	84	31.5
	Any bystander	183	68.5
Self-assessment of participants' knowledge	Very good	63	23.6
	Good	126	47.2
	Poor	78	29.2

AA=Addis Ababa, DRM=Disaster Risk Management

From variables entered into multivariate logistic regression, lack of previous training (p=0.011, AOR=.406(.202-.815), 95%CI), inadequate level of practice (p=.000, AOR=.049(.014-.177), 95% CI) and health service experience below one year (p=.046, AOR=.044(.002-.948), 95% CI) were negatively associated with good level of knowledge (Table 3).

**Table 3:** Multivariate logistic regression to determine factors affecting knowledge level in private general hospitals, AA, July 20-September 30, 2022

Variables	Category	Mean level of knowledge			
		
		Poor N	Good N	AOR CI 95%	P
Marital status	Married/widowed	79	32		
	Single	92	64	1.990(.994-3.982)	.052
Profession	Nurse	62	42	.623(.211-1.840)	.392
	Physician	31	16	.570(.141-2.310)	.431
	Pharmacist/druggist	20	5	.358(.085-1.504)	.161
	Lab technician	35	21	.651(.206-2.059)	.465
	Radiology technician	11	3	.285(.046-1.758)	.176
	Others	12	9		
Educational level	Specialist	22	4	.320(.050-2.049)	.229
	Resident	3	3	1.110(.097-12.690)	.933
	Master/GP	31	21	1.221(.316-4.721)	.773
	BSc	105	58	.806(.258-2.517)	.710
	Diploma	10	10		
Experience (yrs)	<1	8	4	.044(.002-.948)	.046
	1-5	101	60	.092(.007-1.281)	.076
	6-10	54	25	.090(.006-1.265)	.074
	11-15	7	5	.129(.007-2.437)	.172
	16-20	1	2		
Previous training	Yes	27	32		
	No	144	64	.406(.202-.815)	.011
Mean level of practice	Good	3	26		
	Poor	165	70	.049(.014-.177)	.000

AOR=Adjusted Odd Ratio, N=Numbers of respondents, yrs=years, CI=confidence interval, BSc=Bachelor of Science, GP=General Practitioner

**Level of attitude towards disaster risk management**: Majority of the participants believed that every hospital should have disaster risk management plan to handle situations in which there is a sudden large influx of patients (Table 4).

**Table 4:** Assessment of participants' attitude on disaster risk management in private general hospitals, AA, July 20-September 30, 2022

Independent variable	Very much disagree *N (%)*	Disagree *N (%)*	Neutral *N (%)*	Agree *N (%)*	Very much agree *N (%)*
Every health institute should be well prepared to manage disastrous events.	35(13.1)	21(7.9)	20(7.5)	75(28.1)	116(43.4)
Drills should be conducted in every health institution.	19(7.1)	25(9.4)	32(12.0)	94(35.2)	97(36.3)
All frontline HCW should get adequate training to manage patients during disaster.	17(6.4)	22(8.2)	36(13.5)	77(28.8)	115(43.1)
Drills should occur regularly in private general hospitals.	17(6.4)	29(10.9)	49(18.4)	90(33.7)	82(30.7)
Every hospital should have DRMP to handle situations in which there is a sudden large influx of patients.	19(7.1)	16(6.0)	27(10.1)	97(36.3)	108(40.4)
Hospitals should assess the importance of vulnerability.	27(10.1)	22(8.2)	30(11.2)	89(33.3)	99(37.1)
The hospital is unlikely to be affected by disaster	64(24.0)	56(21.0)	46(17.2)	55(20.6)	46(17.2)
Planning for DRM should be given only to the health administrators.	66(24.7)	85(31.8)	34(12.7)	42(15.7)	40(15.0)
DRM is targeted for only doctors and nurses.	76(28.5)	81(30.3)	32(12.0)	42(15.7)	36(13.5)
Disasters are unlikely to happen in our hospital	74(27.7)	63(23.6)	31(11.6)	55(20.6)	44(16.5)
I need to know about disasters and DRM plan	28(10.5)	27(10.1)	30(11.2)	81(30.3)	101(37.8)
Disaster training should be included in health education curriculum.	31(11.6)	32(12.0)	33(12.4)	78(29.2)	93(34.8)

AA-Addis Ababa, DRM=Disaster risk management, N=Number of participants, HCW=Health care workers

This study revealed that overall attitude was positive in 250(93.6%) of the participants.

**Level of practices towards disaster risk management**: In the past one year, 238(89.1%) study participants did not practice what to do on disastrous situation (Table 5).

**Table 5:** Assessment of participants' practices on disaster risk management in private general hospitals, AA, July 20-September 30, 2022

Questions	Responses	Frequency	Percent	Valid percent
In the past one year, have you practiced or drilled on what to do in disastrous situation?	Yes	29	10.9	10.9
	No	238	89.1	89.1
How many drills have you undergone or part already?	One drill	27	10.1	93.1
	2-4 drills	2	.7	6.9
	Total	29	10.9	100.0
Have you participated in ongoing disaster management training in your working private hospital?	Yes	24	9.0	9.0
	No	243	91.0	91.0
How often disaster management training are provided to you within a year?	Once	22	8.2	91.7
	2-4 times	2	.7	8.3
	Total	24	9.0	100.0
Have you seen or heard the disaster plan being periodically updated by authority?	Yes	32	12.0	12.0
	No	235	88.0	88.0
If yes, how often the disaster plan is being updated by authority within a year?	Once per	26	9.7	81.3
	year			
	Twice per	6	2.2	18.8
	year			
	Total	32	12.0	100.0
Have you ever experienced any disaster in your health service stay?	Yes	57	21.3	21.3
	No	210	78.7	78.7
Have you ever been a member of disaster risk management team?	Yes	46	17.2	17.2
	No	221	82.8	82.8
Have you taken first aid training in the past one year?	Yes	88	33.0	33.0
	No	179	67.0	67.0
Do you believe that your practice is sufficient for DRM?	Yes	72	27.0	27.0
	No	195	73.0	73.0

*AA = Addis Ababa, DRM=Disaster Risk Management*

From eligible variables entered to multivariate logistic regression, poor level of knowledge was negatively associated with good level of practice (p=0.000, AOR=.053(.015-.186), 95% CI).

## Discussion

More than 2/3^rd^ (69.7%) of the participants were within of 21-30 years. From the total participants, 139(52.1%) were females while 128(47.9%) were males. The majority of the participants (60.3%) had work experience of 1-5 years. Most of the respondents (77.9%) had never taken training about disaster risk management. This result is comparable with the finding of a similar study conducted in Kenyatta National Hospital (20). However, this result is lower than the finding in Seton Hall University (21), which revealed that 89.4% of participants had prior disaster preparedness training. This may be because of the difference in participants.

**Level of knowledge towards disaster risk management**: This study showed that 171(64%) of the participants had poor level of knowledge regarding disaster risk management, which is supported by reports of studies done in Amhara regional state and AA public hospitals (7, 22). However, it is different from a similar study in Saudi Arabia and Jimma Zone, Ethiopia (23, 24). This discrepancy may be due to the difference in study area.

Only 125(46.8%) participants gave the correct answer about the meaning of disaster risk management. In this study, 82(30.7%) of the participants knew that their institutions had disaster risk management plans, from whom 30(11.2%) knew where they could have found the copy of the plan. This result is different from a study done in AA teaching hospitals where 67% of the participants correctly responded what disaster preparedness is (25) and a result in South Gondar hospitals where 62(41.1%) participants knew the availability of disaster plan in their working institutions (18). From the variables entered into multivariate logistic regression, lack of previous training, inadequate level of practice and health service experience below one year were negatively associated with good level of knowledge. This result is comparable with a the findings of similar study in AA teaching hospitals, Kenyatta National Hospital, and Italian hospitals (20, 25, 26).

**Level of attitude towards disaster risk management**: This study revealed that the overall attitude of the participants was positive in 250(93.6%) of them, which is better than a similar study conducted in frontline nurses in Amhara regional state referral hospitals (7) and KAP of nurses regarding disaster and emergency preparedness in Saudi Arabia (23). From all the participants, 45% of had unfavorable attitude that the hospital is unlikely to be affected by any disaster. This is supported by a comparable study conducted in Central Saudi Arabia (19).

**Level of practice towards disaster risk management**: Only 10.9% of the study participants had adequate practice. This finding is lower than a study done in AA government hospitals in which 56.4% of participants had adequate practice(22). It is also lower than a study conducted in two AA teaching hospitals where 40.6% of study participants had adequate practice(25). The reason behind may be the fact that most private general hospitals did not have disaster risk management plans. Poor level of knowledge was negatively associated with good level of practice, which is in line with a research conducted in AA government hospitals (22, 25).

This study did not include other supportive staff, and the study design was also cross-sectional with limited study area. Occurrence of disaster can happen anytime and anywhere, which mainly affects those with suboptimal preparation. Since involvement of private institutions is important for disaster risk management, HCWs have to be prepared for the proper response.

This study revealed that the majority of the participants had never taken any training about disaster risk management. Only a small proportion of the study participants knew that their hospital had disaster risk management plans. Most of the participants did not know the definitions of disaster, disaster risk management and drill. Although the majority of the participants had positive attitude, the mean levels of knowledge and practice were poor to properly handle large influx of patients. Most of the participants had positive attitude towards the need of disaster training in their health education curriculum and in their working environment. Good level of knowledge was positively associated with good level of practice.
